# On Hospital Gangrene, and Its Efficient Treatment

**Published:** 1865-02

**Authors:** John H. Packard

**Affiliations:** Philadelphia


### ON HOSPITAL GANGRENE, AND ITS EFFICIENT TREATMENT.

By JOHN H. PACKARD, M. D., of Philadelphia.

During the past four months it has been my duty to see and to supervise the treatment of a great number of cases of hospital gangrene, occurring in two of the large military hospitals in the neighborhood of this city. The disorder supervened, in almost every one of these instances, upon gunshot wounds; it presented itself in different grades of severity, and in connection with original injuries varying much in seat and importance. From these extensive observations I have been led to adopt very positive conclusions as to several points in the pathology and treatment of this disease, which I think it but right to lay before the profession.

Let me premise by saying that in so doing it is not my object to claim originality for these views, although some of them have never met my eye in print, nor do I know of their being held by others. But it is very possible that a like experience may have brought some of my fellow-laborers to adopt similar ideas. My own attention was strongly directed to the subject by Lieut.-Col. Le Conte, Medical Inspector, U. S. A., who suggested to me a plan of treatment based upon chemical views. This treatment, simplified as experience showed it could advantageously be, assumed the shape to be presently described.

As every one knows, who has read at all upon military surgery, this disease has prevailed in a most frightful form in the British army, and in some of those of Continental Europe, at different times. Such cases as are described by Blackadder, Boggie, and Hennan, by Guthrie and Macleod, have never yet come under my notice, probably, I think, because of the unquestionably superior *morale* of our army, as well as by reason of the better quality and more regular supply of their food. But no doubt can be entertained of the identity of the disease.

*Phenomena.*—Any open wound may at any time assume this character. A suppurating surface communicating with the atmosphere seems to be a requisite. I have never yet seen an abscess which at the moment of its being opened was the seat of this form of gangrene; nor has it ever occurred to me to see a wholly healed wound break out again with it. Usually the exposure to the air has been of some duration.

Wounds which have healed to a mere point may in a few days spread to several times their original extent. This is most strikingly seen in superficial shell-wounds; in one case of this kind almost the whole outer half of the thigh was thus converted into a ghastly and most offensive ulcer.

When the wound which begins to slough is somewhat wide and shallow, the skin seems to melt away or cave in, as it were, at the edges of the chasm; the epidermis around the margin becoming of a dead pearly white, while the skin below and just outside shows a faint pinkish blush. Another condition is apt to exist in deeper wounds, and especially in sinuses, the connective tissue sloughing so as to undermine the skin, which may or may not look inflamed and unsound.

A grayish-yellow, pultaceous, and horribly offensive material fills up these sores, and flows away like a thick purulent discharge. Microscopically examined, however, it exhibits none of the characters of "laudable" pus; consisting merely of the degenerated connective tissue of the part, with brokendown pus-corpuscles and myriads of minute oil-drops. A few fine fibres, tangled and contorted, are seen, and sometimes here and there a pus-corpuscle less degenerated than the rest.

Swelling is rarely present in a marked degree, unless the wound passes through a fleshy part, such as the arm, calf, or buttock. Another local symptom, especially well pronounced in such cases, is a pungent heat, the temperature being so raised as to be actually quite unpleasant to the hand. If the surrounding atmosphere is at all cool, a steam arises from the surface of the wound. No doubt can be entertained, it seems to me, that this heat is the result simply of the active combustion going on in the part. This rise of temperature I have noticed to be confined strictly to the gangrenous surface. In a wound of large size on the inner side of the thigh, one portion of which was sloughing, while the remainder had been cleaned and was healthy, the difference to the hand held over each part successively was most striking. Pain may be wholly absent, but I have also seen it severe and wearing. The cause of the difference in this respect between different cases I do not know.

It would be difficult to exaggerate the offensiveness of the discharge from wounds in this condition of gangrene. There is a peculiarly nauseous smell over and above that of mere putridity, which I am inclined to attribute to the rancid state of the oil-drops mentioned as seen under the microscope.

One point remains to be noticed, which is the appearance of the surface of these wounds when they are cleaned out by vigorous sponging. A seeming exuberance of pale granulations, often resembling the surface of a bunch of small hydatids, is presented. But so from being exuberant, these granular prominences are merely those parts not yet attacked by the sloughing, and they protrude simply because the tissues between them have been destroyed.

This disorder is strictly local, as has been pointed out by Blackadder and others. It may exist for days, and destroy tissue to a wide extent, without affecting the constitutional condition to any appreciable degree. But this is only the case in men previously robust; generally there is more or less irritative fever, according as the system is more or less readily impressible. Other facts, however, show clearly the local nature of the lesion. One wound may be rapidly spreading and high y offensive, while another in the same limb is healing kind y. Nay, of the same wound one portion may be granulating and filling up, while the remainder of it is sloughing.

Should amputation become necessary, the stump will, if properly made and treated, do as well as in any ordinary case.

When the general system does become implicated, it is by reason of the exhausting effect of the local action, and of pain, and not because there is anything like infection. Perhaps this occurs in some cases, which pass into a typhoid state, but it is not common, and certainly not essential.

*Causes.*—This disease arises epidemically in hospitals, in single wards, or even in separate tents. Having once made its appearance, it spreads rapidly, unless great care is taken in the way to be presently pointed out. It is surely propagated, wherever the same basins or sponges are used indiscriminately for these and for other cases.

But these facts do not account for its origination. No disease should come on more spontaneously, to all appearances, than this often does—none could be more directly traceable to contagion in many cases. It has not yet been observed that any special atmospheric state is apt to attend its appearance, although it seems, as might *à priori* have been expected, that a high temperature favors the putrescent change. Eight or ten very marked cases have, however, occurred under my own notice within the past week (Oct. 20 to Oct. 27) at the Beverly Hospital, among patients in tents, and in a location seemingly free from any possible hygienic disadvantage.

It should be mentioned that the grouping together of grave surgical cases, and especially when these are in any degree overcrowded, seems to favor the breaking out of this affection. But on the other hand this condition may exist without the disease arising, and on the other the disease may be developed where the cases are mild and few. In the spring of 1863, while on duty as a visiting surgeon at the Satterlee Hospital in West Philadelphia, I had under twenty men, all convalescents, in a ward calculated to accommodate fifty, and yet two of the number were suddenly attacked with severe sloughing of their nearly healed wounds.

*Prognosis.*—This is almost always favorable as regards the saving of life—although a patient worn by previous disease might sink under the accession of this new burden. Again, the wound which assumes the gangrenous state may be so situated as not to admit of the needful treatment; penetrating wounds of the chest or abdomen, taking on this condition, have in my experience been uniformly fatal.

This disease may be fatal to a limb which but for it might have been saved; since it lays bare tendons, bones, nerves, and vessels, often involving the two former, especially in necrosis. Hemorrhage is less common in these than would be supposed; it does not necessarily involve amputation, if the artery concerned can be cut down upon and ligated higher up, since the wound so made will do well. Such a course could not be pursued were the disease less purely local.

*Treatment.*—There are two indications always present in cases of this kind. First, all the putrid and putrescent matter must be removed from the wound. Secondly, means must be taken to prevent the recurrence of the sphaccelation or necrosis, into which the tissues will surely run if not checked.

To fulfil the first indication, the forceps are sometimes all that is needed. My own custom is, to seize a portion of the gangrenous connective tissue, and then twist and roll it up, with traction, until it comes away as far as possible, without too great force. Sometimes it will either break or come away; but if it still resists, it must be cut away with a pair of scissors (a bent or curved pair will usually answer best), or with a knife. This must be repeated again and again, until all the tenacious putrilage has been removed. Rough sponging will still further cleanse the surface.

Chloroform or ether should be used in all cases where there is much pain or tenderness in the sore, or when the wound passes deeply through a thick fleshy part, or is undermined at its edges. *The whole surface of the wound should be reached and cleansed;* and the surgeon has not done his work if he stops short of this.

It will do no harm, after this cleansing, to "disinfect" the wound by washing it with the solution of chlorinated soda, with bromine, with a solution of permanganate of potash, or with any other preparation of the kind. But that this is necessary I do not believe, having seen cases in which it was omitted do perfectly well. The thorough cleansing by mechanical means is the main thing to be attended to.

As to the second indication, it is to be met simply by using as a dressing a preventive of oxidation. Sugar, a hydrate of carbon, which does not give up its oxygen, and which is well known for its preservative powers in the case of meats and fruit, is admirable for this purpose. Powdered white sugar is thoroughly and thickly dusted over the wound, or a thick syrup is put on like any other wet dressing, by saturating clean rags with it. I prefer the former method, the sugared surface being covered with wet lint or rags, kept in place by adhesive plaster, or by strips of bandage tied just tightly enough to keep their place.

Coal-oil, turpentine, or any other carbo-hydrogen, if pure, would answer, but the sugar is less offensive, and does not give pain. A mixture of pulverized charcoal with the sugar answers very well when the odor does not quickly disappear after the cleansing.

I believe that wounds still healthy may be prevented from becoming foul and gangrenous from the neighborhood of those which are in the latter state, by the use of sugar or any pure carbo-hydrogen as a dressing, and that the spread of hospital gangrene in a ward may be thus checked.

The cleansing may have to be repeated once, perhaps in some cases twice, before the wound assumes a healthy aspect, but whenever the whole surface can be gotten at the first time this will probably be sufficient. To prevent the spread of the disease by contagion, it is absolutely necessary that each case should have a special sponge and basin set apart for itself, and that these articles should be regularly and thoroughly cleansed after each dressing. Boiling water will effect this.

A few words as to the other plans of treatment in use for this disorder can scarcely be omitted.

Powerful irritants, such as the strong acids or alkalies, or the actual cautery, have been much employed. They depend for any good they may do upon the destruction of the surrounding tissue, and the chance that the ensuing inflammation may not take on a gangrenous form. I have no doubt that in some cases which have come under my observation life has been lost as the consequence of this cruel and random style of practice, although men have recovered in spite of it.

Strong astringents, vegetable or mineral, have been used by some surgeons; the persulphate and perchloride of iron are, I believe, the latest and most favorite of these articles. Although more rational than the former plan, this is an inefficient one in many cases, and is always more or less painful. It is besides much less cleanly than the one I have advocated.

Bromine, so successfully used by Dr. Goldsmith in the Western Military Hospitals is, like chlorine and iodine, a powerful disinfectant. But I can not help thinking that it was *the preliminary cleansing*, described by Dr. G., to which the benefit was really due.

Fermenting poultices are simply nasty, and at the same time useless.

Constitutional treatment can not be expected to check the disease, although it is sometimes indicated by incidental symptoms, and is of course in so far beneficial.

Before closing this paper, it may be right for me to repeat that Dr. Le Conte suggested to me the mode of treatment which has been set forth; his idea was that after the cleansing the permanganate of potash, in solution, should be thoroughly applied to destroy any remaining putrilage, and afterwards the sugar. Practical experience convinced me of the value of this plan, based upon chemical principles; and it was the want of a supply of the solution of the permanganate, which, compelling the omission of the second step of the treatment, proved it to be unnecessary.

In a notice of Dr. Goldsmith's "Report on Hospital Gangrene," etc., by Dr. W. F. Atlee, of this city, published in this Journal for Jan., 1864, the following remarks occur:

"We ourselves have had to treat hospital gangrene, and were entirely satisfied with the results obtained by the following local treatment: The putrid tissues were thoroughly removed; infiltration of the unhealthy secretions among the muscles and under the skin was prevented by the proper application of bandages, and a saturated solution of white sugar was poured upon the sore. We were satisfied with the effect produced by this treatment; we are certain that we should not have any reason to be so at another time, or in other places. Circumstances vary cases of disease as of everything else; they make them curable by simple syrup, or by the application of a simple ointment, or so violent as to defy the actual cautery, and nitric acid, and also, it is most likely, bromine itself."

The difference between these views and those set forth in the preceding paper we conceive to be evident. Remove the existing putrilage, and prevent the formation of more, and the disease is cured. I can not but believe that had my able friend just quoted, followed out his theory and his practice to their full extent, he would have given the plan he so sparingly commends his hearty indorsement. Having been myself an observer of the epidemic of hospital gangrene, which occurred at the Satterlee Hospital in West Philadelphia, in July, 1863, and to which allusion is made in the passage above quoted, I know that the disease as it then presented itself, and that which has come under my notice this summer, are one and the same. And I know that worse cases than have yielded to the simple treatment I have advocated, did not occur in that epidemic; I believe that worse cases could not anywhere be found, unless among men of debauched lives and ruined constitutions, the local poison was supplemented by an utterly depraved systemic state. Under such circumstances, however, we go beyond mere hospital gangrene, which, as has been before argued, is a strictly local disease; it may even be cured in spite of other lesions severe enough to destroy life.

I beg to be excused for again urging the conviction that although hospital gangrene may disappear under the use of the actual cautery, of nitric acid, or of bromine, the happy issue is in such case in spite of the remedy, and not in consequence of it; that the *cure* consists in the removal of all the sloughing and dead tissues, and the *prevention* in opposing oxidation by means of a dressing with any substance which either contains no oxygen or which will not give it up. Once more I must express the entire confidence which a very extended observation has led me to bestow upon this theory, and the resulting line of practice.—*Am. Jour. of Med. Science.*

